# Consultation complexity and professionals consulted: a retrospective cohort study in English primary care

**DOI:** 10.3399/BJGP.2025.0670

**Published:** 2026-05-06

**Authors:** Jialan Hong, Peter Jonathan Edwards, Mavin Nathan Kashyap, Hugh McLeod, Chris Salisbury, Nicola Walsh, Ben Bennett, Isobel L Ward, John Macleod, Christopher Penfold, Maria Theresa Redaniel

**Affiliations:** 1 National Institute for Health Research Applied Research Collaboration West (NIHR ARC West), University Hospitals Bristol and Weston NHS Foundation Trust, Bristol, UK; 2 Population Health Sciences, Bristol Medical School, University of Bristol, Bristol, UK; 3 School of Health and Social Wellbeing, College of Health, Science and Society, University of the West of England, Bristol, UK; 4 Health Innovation West of England, Bristol, UK; 5 National Cancer Registry Ireland, Cork, Ireland

**Keywords:** complexity, general practice, primary health care, workforce

## Abstract

**Background:**

The Additional Roles Reimbursement Scheme (ARRS) launched in England in 2019 to expand the multidisciplinary primary care workforce, but its impact on workload since implementation is unclear.

**Aim:**

To examine changes in workload complexity associated with ARRS implementation.

**Design and setting:**

A longitudinal cohort study was conducted, which used the Clinical Practice Research Datalink (CPRD) Aurum. In total, 3 530 628 consultations were analysed involving GPs, nurses, or direct patient care (DPC)-ARRS roles for 420 986 patients from 369 English practices in 2018 and 2021.

**Method:**

Multilevel logistic regression assessed associations between 17 patient and consultation complexity factors and being seen by a DPC-ARRS role, adjusting for year, age, sex, region, deprivation, and consultation mode.

**Results:**

Complex consultations with DPC-ARRS-eligible roles increased from 15.8% in 2018 to 18.8% in 2021. Diagnostically capable ARRS roles were more likely than GPs to conduct the first consultation after diabetes diagnosis (odds ratio [OR] 1.4, 95% confidence interval [CI] = 1.3 to 1.5) and consultations with ≥2 preventive tasks (OR 5.6, 95% CI = 5.5 to 5.8), but less likely to manage chronic pain (OR 0.8, 95% CI = 0.7 to 0.9), dementia (OR 0.4, 95% CI = 0.3 to 0.4), mental illness (OR 0.4, 95% CI = 0.3 to 0.5), learning disabilities (OR 0.3, 95% CI = 0.3 to 0.4), consultations with ≥3 medicines prescribed (OR 0.6, 95% CI = 0.5 to 0.6), consultations resulted in emergency admission (OR 0.7, 95% CI = 0.6 to 0.8), and consultations with ≥2 diagnoses coded (OR 0.5, 95% CI = 0.5 to 0.5). Patients with interpreter needs (OR 1.2, 95% CI = 1.1 to 1.3), experiencing recent homelessness (OR 1.4, 95% CI = 1.1 to 1.7), or ≥3 long-term conditions (OR 1.1, 95% CI = 1.1 to 1.1) were more likely to be seen by diagnostic ARRS staff.

**Conclusion:**

Following ARRS implementation, primary care activity was delivered by a broader workforce managing increasingly complex care. Further research should assess the safety, quality, and system impacts of ARRS roles.

## How this fits in

The Additional Roles Reimbursement Scheme (ARRS) launched in England in 2019 to expand the multidisciplinary primary care workforce, but its impact on workload complexity is unclear. Using routine records from 369 practices, the study found that direct patient care (DPC)-ARRS roles now deliver a substantially larger share of consultations and that a wider range of clinicians are more active in primary care. Diagnostic capable ARRS roles were more likely than GPs to manage initial diabetes consultations and visits involving multiple routine or preventive tasks. Rising consultation complexity across all professional groups, rather than ARRS role expansion alone, appears to be driving workload changes, highlighting the need for clearer role boundaries, stronger supervision, and effective demand management.

## Introduction

The primary care workforce challenge in England is multifaceted and longstanding. Persistent shortages of GPs and practice nurses exist alongside rising demand and more complex care needs in an ageing, multimorbid population.^1–3^ By 2035, an estimated 67.8% of people aged ≥65 years will live with more than two chronic conditions, further stretching practice resources.^4^

To mitigate these pressures, NHS England introduced the Additional Roles Reimbursement Scheme (ARRS) in 2019 to fund non-medical professionals, diversify teams, improve access and continuity, and bring specialist skills into primary care networks (PCNs).^5,6^ Roles eligible for funding through the ARRS scheme initially included social prescribing link worker, clinical pharmacist, first-contact physiotherapist, physician associate, and paramedic, with other non-medical and medical roles introduced subsequently.^7,8^ Early evaluations have shown large growth in ARRS staffing and appointments, modest gains in access and patient satisfaction, but mixed effects on prescribing, referrals, and quality measures.^9–11^ Furthermore, the scheme has incurred substantial costs, with total spend projected to reach £1.7bn in 2025–2026.^12^

ARRS is intended to give GPs more time to focus on complex consultations and coordination-intensive care by delegating routine tasks (chronic disease reviews, medication checks, preventive care) to other staff.^13–17^ In practice this redistribution operates within the modern general practice model, where reception and clinical staff collect structured information at first contact, prioritise needs, and route patients to the most appropriate professional or service.^18^ However, qualitative and survey evidence reveal unintended consequences; GPs and practice nurses report higher caseload complexity, fragmented care, and workload pressures.^15,17,19,20^ Additionally, pre-labelling presentations as ‘low-complexity’ before a full clinical assessment is difficult and risks mis-triage.^21^ Delivering person-centred care for complex patients therefore depends on effective integration of multidisciplinary teams.

Evidence remains limited on how this diversification of the workforce shapes the management of increasingly complex consultations and diverse patient needs.^22^ To address this gap, this study aims to: 1) describe changes in the proportions of conditions, patient and consultation factors, and complex consultations managed by ARRS roles providing direct patient care (DPC-ARRS), GPs, and nurses before and after ARRS implementation; and 2) examine which complexity factors are associated with the likelihood of being seen by DPC-ARRS roles.

## Method

### Data sources

This longitudinal cohort study used data from the Clinical Practice Research Datalink (CPRD) Aurum, comprising anonymised electronic primary care records from English GP practices using EMIS® GP software. The data were broadly representative of the UK population (CPRD; version 2025.03.001).

The source data included all non-temporary patients (including transferred out and deceased patients) with at least one face-to-face or remote consultation with a GP, practice nurse, or DPC-ARRS role between 1 April 2015 and 31 December 2021 (351 323 923 consultations). Consultations recorded before a practice reached CPRD’s Up To Standard were excluded (*n* = 7 690 861). Consultations with indeterminate or unidentified sex were excluded owing to their extremely low frequency (*n* = 10 989; 0.003%) and potential to affect model convergence. After excluding 265 903 consultations where patients without linkage to 2019 Index of Multiple Deprivation (IMD) data, 343 356 170 consultations remained eligible.^23^ To ensure national representativeness and manageable data volume, 400 practices were randomly sampled, from which 600 000 patients were selected and all their consultations included (*n* = 12 170 079).^9,24^

**Figure 1.**
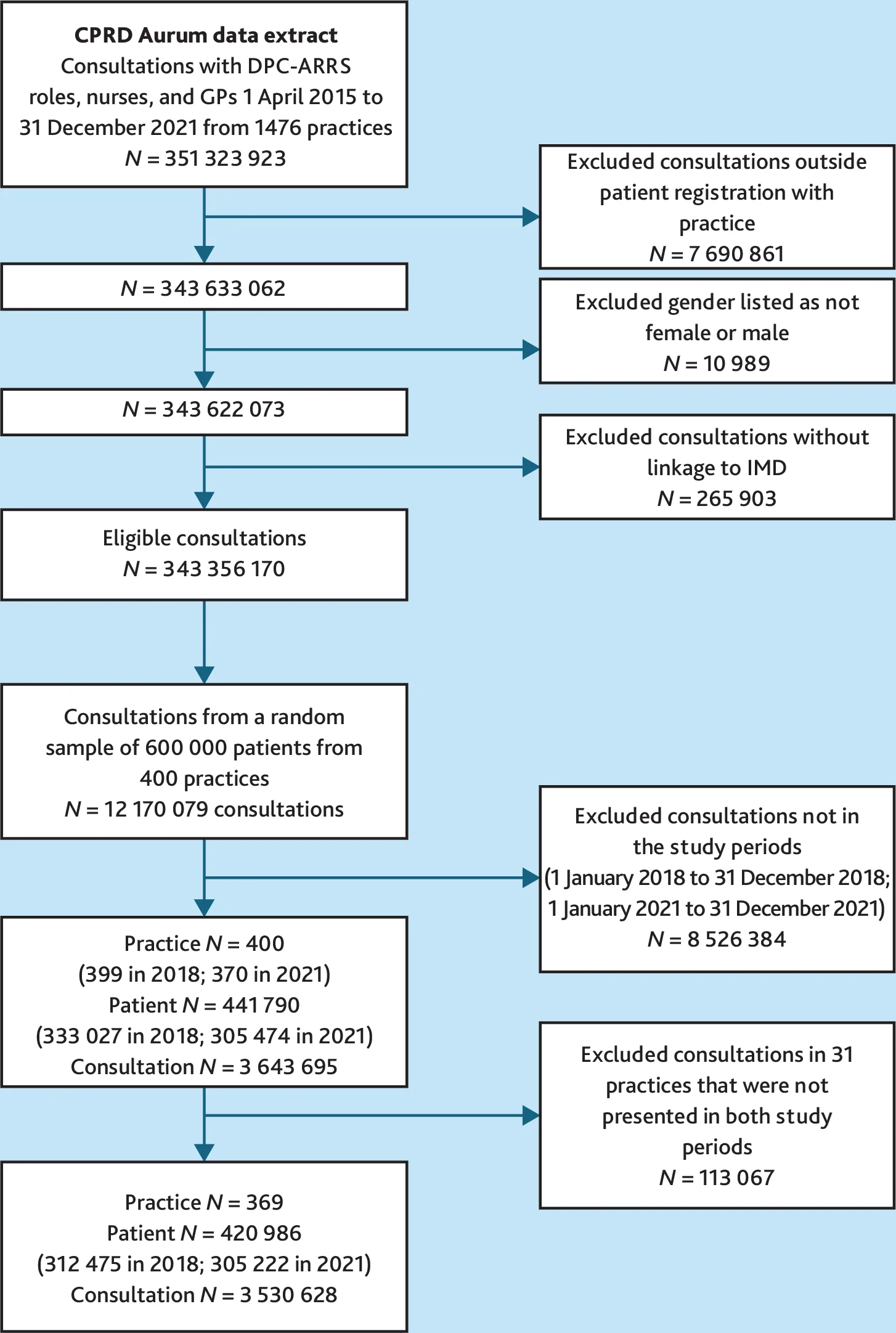
Data flow diagram for selection of samples in Clinical Practice Research Datalink (CPRD). DPC-ARRS = direct patient care-Additional Roles Reimbursement Scheme. IMD = Index of Multiple Deprivation.

This study focuses on the pre- and post-implementation years (2018 and 2021), excluding consultations between January 2019 and December 2020 to minimise COVID-19-related confounding. Of the sampled practices, 369 contributed data in both years, yielding 3 530 628 consultations from 420 986 patients. The sample selection process is illustrated in Figure 1, with practice and patient characteristics presented in Supplementary Table S1. The protocol was approved by CPRD’s Research Data Governance (protocol number: 22_001852).

### Exposures

A published Delphi-validated framework was used that characterised complexity of general practice consultations.^25^ Consultation complexity comprised the following 10 indicators: discussions about drug or alcohol use (C1); chronic pain (C2); dementia (C3); mental health problems (C4); or learning disability or autism (C5); resulting in emergency hospital admission (C6); represented the first consultation following a diabetes diagnosis (C7); involved the prescription of three or more unique substances (C8); included two or more preventive or routine tasks (for example, immunisations, simple chronic-disease reviews, health promotion clinics) (C9); or recorded two or more diagnoses from unique International Classification of Diseases, Tenth Revision (ICD-10) chapters (identified by mapping SNOMED CT codes to ICD-10 codes, C10).

Patient complexity was defined by the following seven factors: a recorded history in the previous year of drug or alcohol abuse (P1); chronic pain (P2); experiencing homelessness (P3); or domestic violence (P4); a recorded need for interpreter services in the past 3 years (P5); three or more major long-term conditions (P6); or a history of personality or disruptive disorder (P7).^25^ A complex consultation was defined as any consultation that involved a patient with any of the seven patient complexity factors or that included any of the 10 consultation complexity factors.^25^

Selected health conditions (ear infection, urinary tract infection, upper respiratory tract infection, and cardiovascular disease) were identified from consultation records. These conditions were chosen in consultation with clinicians and pragmatically selected to represent a range of commonly consulted conditions in general practice. Demographic characteristics were age (categorised as 0–17 years, 18–39 years, 40–64 years, 65−79 years, ≥80 years), sex, region, and area-level deprivation (as defined by IMD).^23,26,27^ Mode of consultation was classified as face-to-face visits (clinic or home) or remote consultations (by telephone, video, and text, web, email based).^9^ The final codelists are available at https://github.com/ARCWest-ADS/ARRS_complexity_codelist/tree/main.

### Clinical roles and grouping

The type of clinical roles that the patient consulted with were GPs, practice nurses, and DPC-ARRS-eligible roles, including chiropodists and podiatrists, dieticians, occupational therapists, paramedics, pharmacists, physician associates, physiotherapists, nursing associates, and mental health practitioners. Advanced nurse practitioners were grouped within DPC-ARRS roles owing to their diagnostic and treatment capabilities and their autonomy in managing patient care, despite not being eligible for ARRS funding until 2023.^28^ Details about the categorisation of GPs, practice nurses, and DPC-ARRS roles have been reported elsewhere.^9^ Diagnostically capable DPC-ARRS roles (paramedics, pharmacists, physician associates, physiotherapists, mental health practitioners, advanced nurse practitioners) were further grouped for role-specific analyses, as these clinicians more commonly manage patients traditionally seen by GPs.

### Statistical analyses

Patient and consultation characteristics were described by role and year and numbers, and proportions of complex patient and consultation factors were reported. To understand the complex cases workload, the proportion of ARRS consultations that were defined as complex consultations were compared with the proportion of GP or nurse consultations.

The authors examined which patient and consultation factors were associated with being seen by six diagnostically capable DPC-ARRS roles. Multivariable multilevel models were constructed with consultations (level 1) nested within patients (level 2) and nested within GP practices (level 3). Role-specific associations were estimated using Firth’s penalised logistic regression to address sparse outcomes. All models adjusted for year, age, sex, region, IMD, and mode of consultation.

### Missing data

With data from electronic health records, absence of a record may reflect non-occurrence or non-recording. Complete-case analysis was used and it was assumed that an absent code indicated the condition was not present.

### Statistical software

All analyses were performed in Stata (version 18.0).

## Results

### Patient characteristics

The authors compared 3 530 628 consultations for 420 986 patients from 369 practices present before (2018) and after (2021) the implementation of ARRS. Table 1 shows that consultations in 2018 were mostly with GPs (68.7%), followed by nurses (22.2%) and DPC-ARRS-eligible roles (9.0%). Between 2018 and 2021, the cohort’s consultation volume increased by 96 440 (5.6%). The proportion of DPC-ARRS consultations increased to 13.1%, while consultations with GPs and nurses slightly decreased to 67.3% and 19.6%, respectively. ARRS involvement increased across all age groups, most notably among older adults (aged 65–79 years: 9.5% in 2018 and 14.4% in 2021; aged ≥80 years: 9.2% in 2018 and 14.4% in 2021). DPC-ARRS consultations increased across all deprivation quintiles, while the two most deprived areas retained lower ARRS consultation rates (12.0% to 12.6%). Between 2018 and 2021, consultations shifted to a predominantly remote model (from 19.3% to 58.6% of all roles). Within face-to-face visits, GPs’ proportion decreased from 65.1% to 49.8%, while nurses’ and ARRS staff’s shares increased to 36.2% and 14.0%, respectively.

**Table 1.** Characteristics of patients at time of consultation by staff role seen, before and after ARRS implementation (***n* = 3 530 628)**

	Before (1 January to 31 December 2018), *n* (%)				After (1 January to 31 December 2021), *n* (%)			
**Variable**	**GP**	**Nurse**	**ARRS**	**Overall**	**GP**	**Nurse**	**ARRS**	**Overall**
**Age group in years**								
0–17	154 007 (68.4)	48 581 (21.6)	22 448 (10.0)	225 036	149 377 (72.0)	35 748 (17.2)	22 334 (10.8)	207 459
18–39	282 349 (72.1)	75 888 (19.4)	33 349 (8.5)	391 586	307 702 (70.9)	73 522 (16.9)	53 012 (12.2)	434 236
40–64	385 391 (70.1)	116 397 (21.2)	47 840 (8.7)	549 628	408 329 (67.2)	118 081 (19.4)	81 158 (13.4)	607 568
65–79	218 624 (63.9)	90 895 (26.6)	32 394 (9.5)	341 913	216 132 (61.8)	83 185 (23.8)	50 478 (14.4)	349 795
≥80	139 460 (66.7)	50 200 (24.0)	19 271 (9.2)	208 931	139 640 (65.1)	44 057 (20.5)	30 779 (14.4)	214 476
**Gender**								
Male	472 600 (68.6)	154 760 (22.5)	61 486 (8.9)	688 846	471 372 (67.5)	135 395 (19.4)	92 028 (13.2)	698 795
Female	707 231 (68.8)	227 201 (22.1)	93 816 (9.1)	1 028 248	749 808 (67.3)	219 198 (19.7)	145 733 (13.1)	1 114 739
**Patient-level IMD quintile**								
1 (Most deprived)	251 947 (67.9)	87 792 (23.7)	31 240 (8.4)	370 979	258 522 (66.8)	79 640 (20.6)	48 668 (12.6)	386 830
2	234 185 (68.4)	81 167 (23.7)	26 901 (7.9)	342 253	244 598 (67.2)	75 503 (20.7)	43 792 (12.0)	363 893
3	226 753 (68.2)	75 493 (22.7)	30 141 (9.1)	332 387	226 443 (66.3)	69 051 (20.2)	46 010 (13.5)	341 504
4	233 580 (69.3)	71 631 (21.3)	31 794 (9.4)	337 005	249 775 (67.7)	68 404 (18.5)	50 790 (13.8)	368 969
5 (Least deprived)	233 366 (69.8)	65 878 (19.7)	35 226 (10.5)	334 470	241 842 (68.6)	61 995 (17.6)	48 501 (13.8)	352 338
**Consultation mode**										
Face-to-face visits (clinic or home)	903 143 (65.1)	358 187 (25.8)	124 984 (9.0)	1 386 314	374 486 (49.8)	271 758 (36.2)	105 411 (14.0)	751 655
Remote contacts (telephone, video, text, web, or email)	276 688 (83.6)	23 774 (7.2)	30 318 (9.2)	330 780	846 694 (79.7)	82 835 (7.8)	132 350 (12.5)	1 061 879
Total	1 179 831 (68.7)	381 961 (22.2)	155 302 (9.0)	1 717 094	1 221 180 (67.3)	354 593 (19.6)	237 761 (13.1)	1 813 534

ARRS = Additional Roles Reimbursement Scheme. IMD = Index of Multiple Deprivation.

### Patient and consultation complexity in consultations with different staff groups

**Figure 2.**
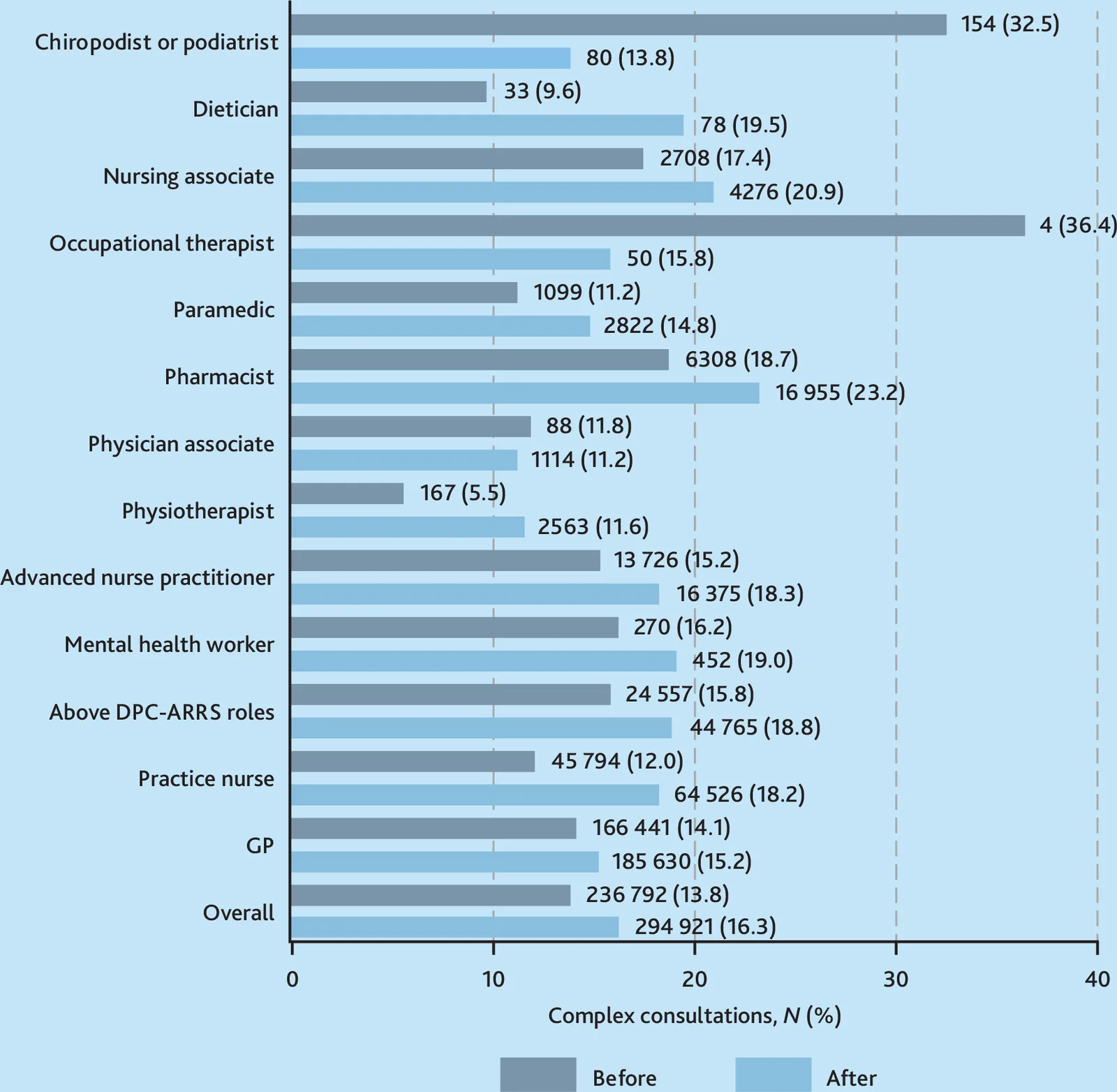
Proportion (normalised) of consultations seen by each staff role that were complex, before and after ARRS implementation (*n* = 3 530 628). DPC-ARRS = direct patient care-Additional Roles Reimbursement Scheme.

All three groups of staff — GPs, nurses, and DPC-ARRS — experienced an increase between 2018 and 2021 in the proportion of their consultations that were complex (Figure 2). Practice nurses experienced the greatest increase (from 12.0% to 18.2%), followed by DPC-ARRS roles (from 15.8% to 18.8%) and then GPs (from 14.1% to 15.2%). Most DPC-ARRS roles had an increased proportion of complexity consultations, except for chiropodists or podiatrists, occupational therapists, and physician associates.

DPC-ARRS activity increased across most patient and consultation factors (Table 2): consultations about cardiovascular disease (from 8.2% to 15.7%), about chronic pain (8.5% to 17.3%), about drug or alcohol abuse (6.5% to 15.8%), and with two or more recorded diagnoses from unique diagnostic chapters (5.7% to 11.7%). DPC-ARRS consultations with complex patients also increased: interpreter needs in the past 3 years (8.8% to 14.5%), drug or alcohol abuse in the previous year (8.8% to 14.1%), and chronic pain in the previous year (9.9% to 15.7%).

**Table 2.** Distribution of consultation and patient factors by staff roles seen, before and after ARRS implementation (*n* = **3 530 628**)

	ARRS implementation *n* (%)							
	**Before (1 January 2018 to 12 December 2018)**				**After (1 January 2021 to 12 December 2021)**			
	**GP**	**Nurse**	**ARRS**	**Overall**	**GP**	**Nurse**	**ARRS**	**Overall**
**Consultations about:**								
Cardiovascular disease (CVD)	5937 (71.3)	1700 (20.4)	684 (8.2)	8321	4225 (65.9)	1178 (18.4)	1008 (15.7)	6411
Ear infection	8269 (82.4)	294 (2.9)	1469 (14.6)	10 032	4716 (78.1)	224 (3.7)	1096 (18.2)	6036
Urinary tract infection (UTI)	5045 (89.0)	113 (2.0)	511 (9.0)	5669	2520 (88.6)	69 (2.4)	255 (9.0)	2844
Upper respiratory tract infection (URTI)	26 794 (85.7)	687 (2.2)	3780 (12.1)	31 261	12 557 (83.2)	337 (2.2)	2190 (14.5)	15 084
Drug or alcohol abuse (C1)	3346 (81.1)	514 (12.5)	267 (6.5)	4127	2801 (68.1)	661 (16.1)	652 (15.8)	4114
Chronic pain (C2)	1866 (90.3)	25 (1.2)	176 (8.5)	2067	1830 (81.0)	38 (1.7)	390 (17.3)	2258
Dementia (C3)	3590 (88.8)	182 (4.5)	270 (6.7)	4042	2607 (83.1)	188 (6.0)	343 (10.9)	3138
Mental illness (C4)	2314 (89.2)	182 (7.0)	99 (3.8)	2595	1931 (80.5)	235 (9.8)	232 (9.7)	2398
Learning disability or autism (C5)	1181 (80.8)	162 (11.1)	119 (8.1)	1462	1397 (69.1)	416 (20.6)	209 (10.3)	2022
Result in emergency hospital admission (C6)	1266 (85.3)	46 (3.1)	172 (11.6)	1484	682 (84.5)	31 (3.8)	94 (11.6)	807
First consultation after diabetes diagnosis (C7)	1981 (57.5)	981 (28.5)	486 (14.1)	3448	1564 (54.6)	773 (27.0)	527 (18.4)	2864
≥3 unique substances prescribed (C8)	13 224 (90.9)	289 (2.0)	1033 (7.1)	14 546	7323 (88.8)	113 (1.4)	807 (9.8)	8243
≥2 preventive or routine tasks carried out (C9)	11 140 (32.4)	16 606 (48.4)	6584 (19.2)	34 330	9340 (22.1)	23 529 (55.7)	9351 (22.1)	42 220
≥2 diagnoses from unique chapters recorded (C10)	39 534 (90.2)	1791 (4.1)	2516 (5.7)	43 841	22 383 (81.5)	1859 (6.8)	3224 (11.7)	27 466
**Consultations with patients had:**								
Drug or alcohol abuse in previous year (P1)	17 717 (75.0)	3805 (16.1)	2086 (8.8)	23 608	19 819 (70.6)	4299 (15.3)	3957 (14.1)	28 075
Chronic pain in previous year (P2)	12 334 (76.2)	2238 (13.8)	1605 (9.9)	16 177	14 394 (72.6)	2326 (11.7)	3113 (15.7)	19 833
Experiencing homelessness in previous year (P3)	730 (67.8)	140 (13.0)	207 (19.2)	1077	751 (72.1)	126 (12.1)	164 (15.8)	1041
Domestic violence in past year (P4)	1970 (82.0)	304 (12.7)	127 (5.3)	2401	2280 (78.0)	331 (11.3)	313 (10.7)	2924
Interprete needed in past 3 year (P5)	5024 (74.5)	1128 (16.7)	590 (8.8)	6742	5693 (71.6)	1110 (14.0)	1151 (14.5)	7954
≥3 long-term conditions (P6)	76 139 (66.9)	25 016 (22.0)	12 634 (11.1)	113 789	116 154 (64.1)	37 680 (20.8)	27 275 (15.1)	181 109
Personality or disruptive disorder (ever) (P7)	4567 (83.8)	348 (6.4)	534 (9.8)	5449	7827 (75.9)	952 (9.2)	1536 (14.9)	10 315

ARRS = Additional Roles Reimbursement Scheme.

**Figure 3.**
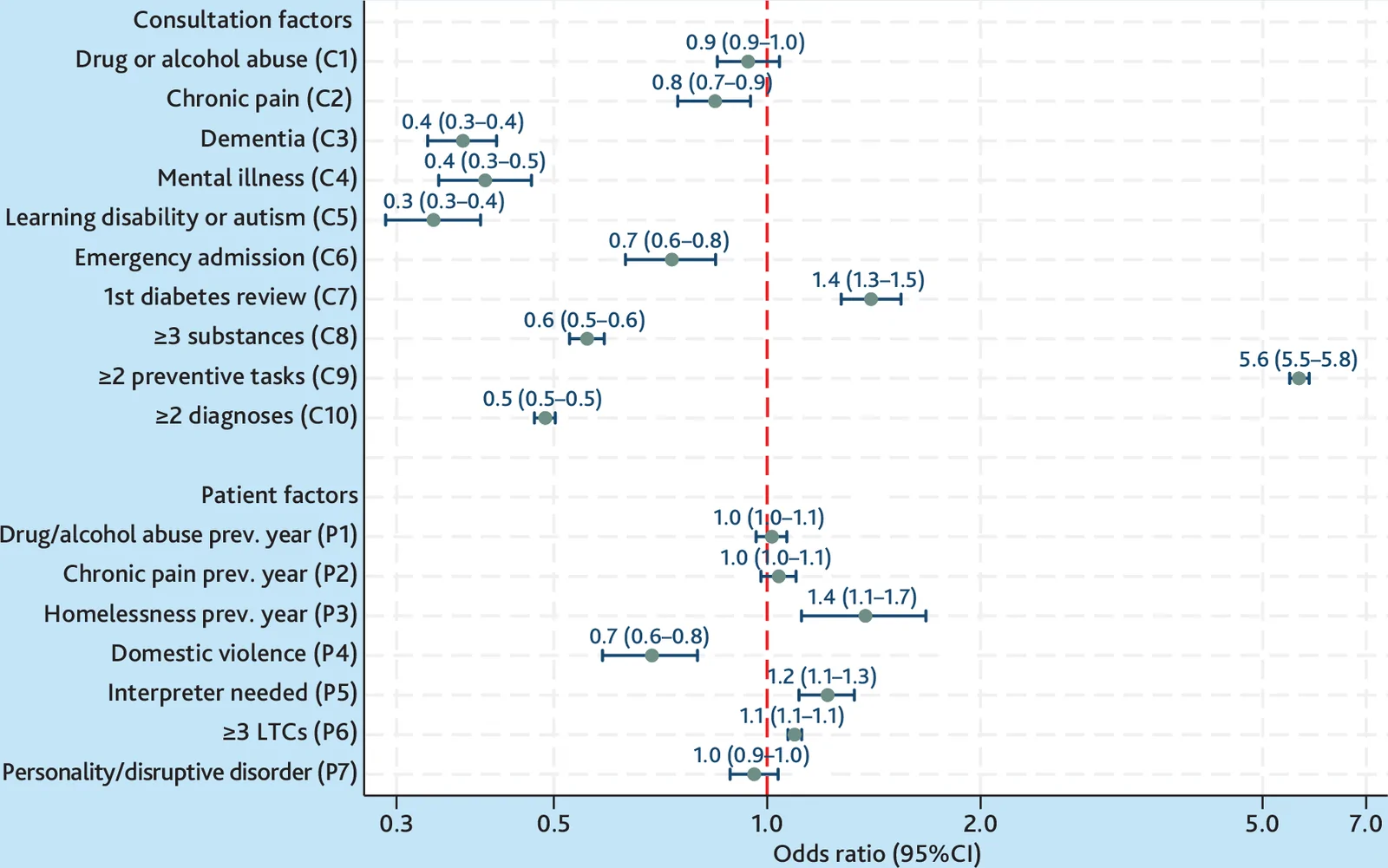
Associations between consultation and patient complexity factors and being seen by direct patient care-Additional Roles Reimbursement Scheme (DPC-ARRS) roles with diagnostic capabilities in general practices in England (*n* = 2 756 314). Note: multivariable model adjusted for all complexity factors, year (before or after ARRS implementation), age group, sex, Index of Multiple Deprivation (IMD), and region simultaneously. Odds ratios (ORs) with 95% confidence intervals (CI) were log-scaled. ORs >1 indicate greater odds of seeing a DPC-ARRS role with diagnostic capabilities rather than a GP. OR for time period (year) = 2**.**2, 95% CI = 2.2 to 2.3. OR for mode of consultation = 0.6, 95% CI = 0.6 to 0.6. Random-effects parameters: var(practice) = 2.9, 95% CI = 2.5 to 3.5; var(patient) = 1.4, 95% CI = 1.4 to 1.5). LTCs = long-term conditions. Prev. = previous. var = variance.

### Consultation complexity factors of ARRS roles compared with GPs

The study found that diagnostic ARRS roles were more likely than GPs to manage first consultations after diabetes diagnosis (odds ratio [OR] 1.4, 95% confidence interval [CI] = 1.3 to 1.5), which were driven by advanced nurse practitioners (OR 2.1, 95% CI = 1.9 to 2.3) and pharmacists (OR 1.8; 95% CI = 1.6 to 2.1); and consultations with two or more preventive or routine tasks (OR 5.6; 95% CI = 5.5 to 5.8), a pattern present across most diagnostic roles (ORs: 1.2–5.0), except physiotherapists. DPC-ARRS roles were less likely than GPs to manage chronic pain (OR 0.8, 95% CI = 0.7 to 0.9), dementia (OR 0.4, 95% CI = 0.3 to 0.4), mental illness (OR 0.4, 95% CI = 0.3 to 0.5), learning disabilities (OR 0.3, 95% CI = 0.3 to 0.4), consultations with ≥3 medicines prescribed (OR 0.6, 95% CI = 0.5 to 0.6), consultations resulted in emergency admission (OR 0.7, 95% CI = 0.6 to 0.8), and consultations with ≥2 diagnoses coded (OR 0.5, 95% CI = 0.5 to 0.5) (Figure 3, Supplementary Table S2).

Different roles showed clear specialisations (Supplementary Table S2). Pharmacists and mental health practitioners managed more drug and alcohol-related cases; pharmacists and physiotherapists saw more chronic pain consultations; physician associates and mental health practitioners saw more dementia; paramedics had higher subsequent emergency admissions; mental health practitioners delivered more mental illness consultations; and physician associates handled more learning disabilities or autism cases. GPs remained more likely than any diagnostic roles to manage polypharmacy (three or more unique prescriptions) and consultations coded with multiple diagnoses chapters.

### Patient complexity factors of ARRS roles compared with GPs

Diagnostic ARRS roles were more likely than GPs to see patients experiencing homelessness (OR 1.4, 95% CI = 1.1 to 1.7), patients needing interpreter services (OR 1.2, 95% CI = 1.1 to 1.3), and patients with three or more long-term conditions (OR 1.1, 95% CI = 1.1 to 1.1). Pharmacists and mental health practitioners saw more patients with recent substance problems; pharmacists, physiotherapists, and mental health practitioners saw more with chronic pain; pharmacists and advanced nurse practitioners saw more with a history of experiencing homelessness; mental health practitioners saw more domestic violence cases; and pharmacists and mental health practitioners saw more personality or disruptive disorder cases (Figure 3, Supplementary Table S2).

## Discussion

### Summary

Following ARRS implementation, the contribution of DPC-ARRS roles to primary care consultations increased. Increases were greater in more deprived areas, although absolute ARRS consultation rates remained higher in less deprived quintiles by 2021. There was a substantial increase in the proportion of consultations managed by nurses and DPC-ARRS roles that were complex and a small increase in the proportion of GP consultations that were complex. DPC-ARRS roles, especially those with diagnostic capacity, now manage a broader mix of complex and non-complex tasks, notably first consultation following a diabetes diagnosis, multimorbidity, and preventive or routine visits. More than half of flagged complex consultations involved multiple long-term conditions, explaining much of this trend.

### Strengths and limitations

Strengths include the large, routinely collected dataset spanning multiple practices at two discrete time points and robust statistical methods. However, this study has several limitations. ARRS-eligible roles cannot be comprehensively identified owing to variation in staff coding within primary care records, particularly those for care coordinators and social prescribing link workers.^9^ For some roles with relatively few consultations, effect estimates may be inflated despite using Firth’s penalised regression to address sparse-data bias. The analysis reflects the early ARRS rollout and does not capture later role expansions of the scheme, such as nurses, newly qualified GPs, health support workers, and other advanced practitioners. Moreover, role and consultation modality coding in routine data are imperfect, potentially misrepresenting general practice workload and resource allocation, particularly for virtual consultations.^29^ Furthermore, to robustly measure within-practice change, the longitudinal analysis was restricted to 369 practices contributing data in both years. It may limit generalisability, especially to smaller or more vulnerable practices that ceased contributing data. This attrition reflects a known national decline in practice numbers and should be considered when interpreting the findings.^30^

The definition used of ‘complex consultation’ in this article was based on a Delphi study among GPs and validated against the duration of GP consultations.^25^ Accordingly, it is more applicable to consultations historically seen by a GP and may align better with diagnostically capable ARRS staff than other roles. For example, a nurse dressing change may not reflect GP-derived complexity factors. Nurses or ARRS staff may perceive complexity differently, and our measure may not capture all dimensions of complexity, particularly emerging social and digital health factors, and may under- or overestimate complexity in certain consultations.^22^ Additionally, given the diversity of tasks undertaken by ARRS staff, grouped comparisons, even when limited to diagnostically capable roles, may be too crude. Role-specific associations are reported, but precision is limited for some roles (for example, physician associates) owing to small sample sizes.

Furthermore, clinical decisions regarding allocation of patients to staff may be influenced by unmeasured factors, such as clinician availability, practice-level protocols, or patient preferences. Some of the overall increase in complexity is likely owing to an ageing population, but it may also reflect improved coding of primary care activities over time. Consultation complexity may be shaped by the staff member consulted, rather than determining who the patient is seen by. These factors should be considered when interpreting the findings.

### Comparison with existing literature

The rise in complex consultations across all professional groups contrasts with expectations and stakeholders’ views that ARRS roles would absorb simpler tasks, enabling GPs to focus on more clinically challenging care.^2,31,32^ Instead, this study’s findings suggest parallel trends: increasing complexity for GPs and nurses alongside an expanded clinical scope among diagnostically capable ARRS staff. These roles now contribute advanced expertise in multimorbidity management and medication reviews (pharmacists), holistic long-term care (advanced nurse practitioners), and allied health management of chronic conditions (for example, physiotherapists, chiropractors, and podiatrists).^33–35^

These patterns likely reflect broader system pressures rather than ARRS expansion alone. Population ageing and post-COVID service recovery have concentrated work into fewer, more complex appointments, as deferred face-to-face reviews and vaccination activity increased workload across staff groups.^36–38^ Practice nurses experienced the largest increase in complexity, consistent with previous evidence that the ARRS implementation can duplicate nursing workload and introduce additional unfunded supervisory and ‘safety-netting’ responsibilities as protocol-driven tasks shift to DPC-ARRS staff.^20^

Qualitative and ethnographic studies provide further context, highlighting role ambiguity, conflicting accountabilities, and variation in employment models during ARRS implementation.^2,17,20,32,39^ Together, this evidence suggests that primary care has been reshaped through a combination of task redistribution, organisational adaptation, and rising patient complexity, rather than a simple substitution of GP work.

Moreover, ARRS consultations grew fastest in more deprived areas with higher needs, consistent with previous observation.^17,40^ However, the absolute proportions remained higher in the less deprived areas. This suggests that deprivation-targeted expansion has not overcome baseline capacity advantages, such as greater workforce stability and infrastructure in less deprived areas.

### Implications for research and practice

The concurrent growth in ARRS activity and complex consultations across all professional groups points to redistribution of work within an increasingly pressured system. While ARRS has broadened capacity and skill mix in primary care, rising consultation complexity appears to be a system-wide shift driven by ageing populations, deferred care, and post-pandemic recovery, rather than ARRS expansion alone.^22^ Simply increasing role numbers is therefore unlikely to reduce GP workload.

With the recent addition of experienced nurses and newly qualified GPs, PCNs should clarify role boundaries, implement standard referral and handover protocols, strengthen supervision and mentorship, and ensure adequate administrative support, especially in deprived areas.^2,17,41^ Workforce‐diversification strategies must be paired with robust demand‐management measures, including enhanced triage protocols and related supervision, to ensure that expanded capacity translates into addressing unmet need rather than inadvertently increasing workload.^42^ These strategies must be implemented with careful oversight to maintain quality, coordination, and continuity of care. Policymakers should recognise that many ARRS practitioners now routinely undertake complex, patient-centred tasks, and future funding, training, and career frameworks must reflect these advanced responsibilities.

Caution is also warranted when relying on ARRS staff to manage complex patients and problems, given their more limited and circumscribed training than GPs. Evidence on the safety, effectiveness, and appropriate scope of ARRS roles in managing complex cases remains limited.^43^ Further research, particularly longitudinal health-economic evaluations and intervention trials, is needed to assess downstream impacts on service utilisation, potential demand generation, and how demand-management strategies can prevent further concentration of complexity within GP and nursing workloads.

## Data

All data relevant to the study are included in the article or uploaded as supplementary information in the appendix. Raw data from the study are not publicly available. Data for the study were obtained under licence from the Clinical Practice Research Datalink (CPRD); pseudonymised patient data are available from CPRD subject to Research Data Governance approval; see https://www.cprd.com/how-access-cprd-data for more information.
